# Materia Medica: What Is Needed

**Published:** 1887-12

**Authors:** 


### MATERIA MEDICA: WHAT IS NEEDED.

EDITORS HOMŒOPATHIC PHYSICIAN: Page 369 of your October issue, the symptom, "catamenia only in the morning, Sepia," is credited to Hering. We cannot find it in the *Condensed* nor in symptoms of the mind (Hering's *Analytical*). Allen gives Hahnemann as the authority, and as the symptom is not found in Hempel's translation of the *Chronic Diseases*, it proves that a retranslation of this great work is a necessity. We members of the I. H. A. could not do a greater service to our school than by undertaking this work. I, for one, would be willing to do my share toward it if a revisory board could be selected so we may be sure that the translation is correct. It is sometime very difficult to get the true meaning of Hahnemann's German.

In fact, we doubt the correctness of the symptom, "catamenia only in the morning," which may mean at no other time, day or night, or it may mean any hour after midnight or in the morning before or after rising. I believe in reading between the lines, as Professor Jones, of Ann Arbor, used to say; this symptom may read "great weakness in morning during menses," which would well agree with the profuse menstruation and the relaxation of the pelvic organs.

We do not need a condensed *Materia Medica*, but we do need a *master mind* to explain to us the character of a remedy given, that then the other symptoms group around it as do the stars around the sun.

Fraternally, S. L.

[Dr. Hempel was generally a very careless translator and editor, but as regards this Sepia symptom he is wrongly accused, as the symptom is given in *Chronic Diseases*, vol. V, p. 171, line 15. Yet so unreliable was Dr. Hempel, as a rule, that we can cordially indorse S. L.'s proposition for a careful revision of Hahnemann's works. It is (and has always been) a misfortune that the English student has access to so many of Hahnemann's works through such unreliable translations. At the last meeting of the I. H. A., Dr. John Hall, of Toronto, proposed a new translation of the *Organon*—another "long-felt want."

As to this Sepia symptom, Jahr, Hahnemann (*Chronic Diseases*), Lippe, and Guernsey give it as menses appearing only in the morning. The symptom, as Jahr (*Symptomen Codex*, vol. II, p. 782), gives it, reads: "Menses too early by eight days, and too scanty, appearing only in morning." The Sepia menstruation is rather more apt to be scanty than profuse, hence, "reading between lines," we might say the menses are too early and *so* scanty as to show only part of the time, that is, only in the morning. Dr. Minton gives the symptom wrongly, for "generally too early and *too profuse*" read *too scanty*.]

